# Adapting the 2022 WHO verbal autopsy tool for use in Lagos State, Nigeria: insights from the LVASA-SRS project

**DOI:** 10.1186/s13104-025-07560-1

**Published:** 2025-12-29

**Authors:** Adeyemi Okunowo, Uchenna Gwacham-Anisiobi, Ndubuisi Ezumezu, Hameed Adelabu, Adedoyin Ogunyemi, Teniola Lawanson, Brenda Isikekpei, Bosede B. Afolabi, Aduragbemi Banke-Thomas

**Affiliations:** 1 Centre for Clinical Trials, Research, and Implementation Science, College of Medicine, University of Lagos, Idi Araba, Lagos State Nigeria; 2 Faculty of Epidemiology and Population Health, London School of Hygiene and Tropical Medicine, Keppel St, London, WC1E 7HT UK; 3 Department of Obstetrics & Gynaecology, College of Medicine, University of Lagos, Idi Araba, Lagos State Nigeria; 4 Department of Obstetrics & Gynaecology, Lagos University Teaching Hospital, Idi Araba, Lagos State Nigeria; 5 Department of Community Health and Primary Care, College of Medicine, University of Lagos, Ikeja, Lagos State Nigeria

**Keywords:** Maternal mortality, Stillbirth, Verbal autopsy, Adaptation, Nigeria, Public health surveillance, Health systems

## Abstract

**Objective:**

Accurate and routine cause-of-death data are essential for health planning, yet many low- and middle-income countries lack comprehensive systems for such data, resulting in undocumented maternal and perinatal deaths. This article details the adaptation of the 2022 WHO Verbal Autopsy (VA) tool for maternal mortality surveillance in a linguistically diverse urban setting with high rates of out-of-facility deaths as part of the Lagos Verbal and Social Autopsy Sample Registration System project in Nigeria.

**Results:**

The adaptation process enhanced the tool’s cultural sensitivity, language clarity, and system compatibility. Impersonal or potentially disrespectful terms (e.g., “the deceased”) were replaced, complex medical terms simplified (e.g., “abdominal pain” to “tummy pain”), and locally relevant options added for care providers and death locations. Context-specific skip patterns improved efficiency by focusing on maternal deaths and a rigorous translation process (forward/back translation in Yoruba and Pidgin English) ensured linguistic fidelity and local resonance. Validation included face and content review, role-play simulations, and pilot-testing with bereaved families. The adapted tool improved respondent comfort, data accuracy, and digital integration, incorporating global positioning system coordinates and interviewer identification numbers. The adapted tool retained compatibility with automated VA coding platforms and was incorporated into training regimen for data collectors.

**Supplementary Information:**

The online version contains supplementary material available at 10.1186/s13104-025-07560-1.

## Introduction

Accurate and timely data on mortality and causes of death (CoD) are fundamental for public health planning, evaluation, and policymaking. However, many low- and middle-income countries (LMICs) lack robust civil registration and vital statistics (CRVS) systems capable of providing comprehensive, routine mortality data. Contributing factors include inadequate financing, poor infrastructure, lack of intra- and inter-agency collaborations, weak legal frameworks, and contextual sociocultural barriers [1, 2]. In these settings, a significant proportion of deaths occur at home or outside health facilities and remain undocumented or uncertified, limiting the ability to monitor disease burdens, allocate resources, or assess progress toward global health targets [3]. 

To address this, the World Health Organization (WHO) recommends the use of verbal autopsy (VA) within the framework of Maternal and Perinatal Death Surveillance and Response (MPDSR), as a method of data collection that involves interviewing caregivers or relatives of the deceased to gather information about symptoms and circumstances preceding death [4, 5]. Responses are used to infer probable causes of death, through physician review or automated coding tools [5, 6]. VA is increasingly used in LMICs as a simple, low-cost, and relatively efficient alternative for collecting vital statistics on population mortality, particularly where conventional CRVS systems are weak or absent [4, 7]. 

VA has historical roots in community-based death investigation, with the term first used during the Narangwal Project in India in 1956 [5, 6]. Its formalisation accelerated in the 1950s–60s, with structured interviews by physicians in Asia and Africa. WHO later promoted lay health reporting and developed simplified instruments [5]. However the proliferation of disparate VA tools raised concerns over validity and comparability, prompting the development of a harmonised WHO standard [5, 6]. The first WHO International VA standard tool was developed in 2007 and has been updated in 2012, 2014, 2016, and 2022 [5, 8] to improve usability, reflect advances in data collection and analytics, and enable integration with health systems [5, 6]. These updates have made the tool suitable for research, by generating reliable and comparable CoD estimates, and for policymaking, by providing timely and actionable data [5]. 

WHO promotes VA as an interim solution in CRVS-deficient settings, and while the tool is designed to be globally applicable and necessarily generic, it requires local adaptation [4, 5, 9]. Inappropriate terminology, language barriers, and cultural misalignment can undermine data quality and respondent cooperation. WHO, therefore, provides structured guidance to ensure adaptations maintain the instrument’s validity and comparability while addressing local needs [9]. A summary of these recommendations is provided in Table 1. However, despite the availability of such guidance, few LMIC studies have documented the practical process of adapting the tool to local linguistic and cultural contexts. Although the tool has been used in a few Nigerian studies, [10–12] none have detailed how the adaptation was undertaken or what challenges were encountered. This lack of documentation represents a critical gap, particularly in settings with persistent challenges in maternal mortality and diverse sociocultural contexts. The objective of this article is to address this knowledge gap by documenting the process of adapting the 2022 WHO VA tool for maternal mortality surveillance in Lagos State, Nigeria, a linguistically and culturally diverse setting. By so doing, we provide a model for contextualising VA in LMICs, balancing standardisation with sensitivity to local realities.

**Table 1:** Guidelines for adaptation of the 2022 WHO verbal autopsy tool

SN	Recommendations
1.	Only changes to the wording of existing variables to enhance local comprehension or ensure cultural acceptability of questions should be undertaken.
2.	Need and rationale for modification should be shared with WHO.
3.	Questions may be added, but no questions should be removed from the original list because of the negative impact on the comparability of the CoD information and the future data-based evolution of the instrument.
4.	Age, sex, information about the season, the local prevalence of HIV and malaria, and Sects. 3, 5, 6 and 7 are essential information for the analytical software that assigns CoD. As a result, no questions must be removed from these sections, and the numbering of the questions must remain unchanged. Questions added locally will not be used by the analytical software.
5.	Adding “signs and symptoms” to the 2022 WHO VA instrument needs to be carried out with great caution as alteration can compromise the comparability of VA data between populations.
6.	Modifications may be necessary if there are emerging or locally important CoD for which there are no questions on the 2022 WHO VA questionnaires. In these circumstances, advice may be sought from WHO for making such modifications. If modifications are necessary, they should be carefully documented and distinguished from the 2022 questionnaire sections and variables.
7.	Adding questions or sections about household characteristics, environmental or behavioural risk factors, and adding or changing questions about usage of a particular health context are unlikely to affect the comparability of results.
8.	Changing or adding to response categories in the checklist of “signs and symptoms noted during the final illness”; or adding new questions about diseases of particular interest (e.g., malaria, HIV/AIDS, diarrhoea disease) may affect the comparability of results.
9.	Adding and removing questions may compromise the usability of analytical software for assigning CoD. It may either not be possible to use the existing analytical software for assigning the newly added CoD, or the outputs from the software may become unreliable.
10.	During translation, the notes in the Table of Indicators and the “Question by Question” descriptions in the “VA Field Interviewer Manual” should be translated, because the notes provide guidance to interviewers.
11.	For quality assurance, a second translator should carry out a back-translation to English after initial forward translation to the local language.
12.	It is important for both interviewers and respondents to understand the medical terminology used in the VA questionnaire. Care should be taken to ensure that medical terms do not lose their meaning when translated into the local languages.
13.	It may be useful to develop a list of common terms locally used to capture the medical terminology in the VA questionnaire. These should be used during training for interviewers and during the VA data collection.

## Main text

The 2022 WHO VA tool for adults consists of seven sections, namely: preset questions on Human Immunodeficiency Virus, malaria, mortality, and seasonality; information about the respondent and background to the interview; details about the deceased and vital registration; an open narrative section; history of injuries or accidents; and medical history related to the final illness. The final section consists of a detailed health history including the duration of illness, general signs and symptoms associated with the final illness, signs and symptoms associated with pregnancy and women, signs and symptoms, health service utilisation, civil registration numbers and death certificate with cause of death [5]. Adaptation is essential to ensure the tool remains valid while being culturally and contextually appropriate, with careful consideration needed of its acceptability within local norms, beliefs, and practices [13]. 

### Setting

According to the Lagos State Government, the state, which is Nigeria’s most populous, had an estimated population exceeding 27 million people and a population density of 7,390 persons per km^2^ as of 2020 [14]. It is a metropolitan and highly urbanised state characterised by multiculturalism, multilingualism, and significant socio-economic heterogeneity. Despite its economic prominence, Lagos grapples with public health challenges, particularly in underserved informal settlements where institutional births are low, and home deaths including maternal deaths are common.

The adaptation of the 2022 WHO VA tool was carried out as part of the Lagos State Verbal Autopsy and Social Autopsy Sample Registration System (LVASA-SRS) initiative, a project aimed at generating accurate maternal mortality ratio and stillbirth rate estimates and establishing a system for routine mortality surveillance. The project prioritised adapting the VA tool to local contexts to ensure high-quality data collection and community acceptability.

### Adaptation process

The objectives of the adaptation process were to enhance question clarity, improve comprehension among interviewers and respondents, and ensure cultural and linguistic appropriateness. Adaptation occurred in three phases: (1) content modification, (2) language translation and (3) rigorous quality testing to preserve the tool’s validity and reliability. The process followed WHO guidelines [5] (Table 1) and the methodological framework proposed by Sousa et al. [9].

#### Content modification

Modifications to the tool were guided by the need to enhance clarity, cultural appropriateness, and alignment with local health system realities. Adaptations were informed through a consultative process involving local public health experts, maternal health professionals, and individuals with deep contextual and cultural knowledge. These stakeholders reviewed the content for linguistic accuracy, sociocultural sensitivity, and conceptual clarity to ensure relevance and acceptability within the Lagos State context. Specific changes were related to:

1. *Cultural appropriateness* Some questions and response options in the VA tool conflicted with local cultural norms. For instance, the “ambiguous/intersex” category was removed following stakeholder advice that such cases are extremely rare in maternal mortality contexts and that its inclusion could cause confusion or discomfort for respondents. To minimise exclusion bias, interviewers were trained to sensitively probe for pregnancy history regardless of gender presentation, and narrative fields were retained to capture relevant details outside fixed response categories.

In Nigeria, communication with the bereaved prioritises empathy, politeness, honour and respect especially when referring to the deceased. The term “the deceased” was considered impersonal and was replaced with personal pronouns such as “she” or “her” when discussing maternal deaths. More generally, wording perceived as disrespectful or likely to offend was revised to reflect culturally appropriate language.

2. *Language appropriateness* Some terms in the VA tool were unfamiliar to respondents due to variations in literacy levels and socio-cultural backgrounds, which limited comprehension and hindered effective communication. For instance, technical phrases such as “road transport injury” were not well understood by many respondents and were replaced with more commonly used expressions like “road traffic accident” to enhance clarity and engagement.
3. *Simplification of medical terminologies* Complex medical terms were replaced with simpler, widely understood ones to aid respondents with limited medical knowledge. For instance, “abdominal pain” became “tummy pain”, “extreme fatigue” was simplified to “always tired”, and “puffiness” to “swollen”.
4. *Reflecting contextual variations in health system utilisation and death locations* The tool was revised to include types of care providers and death locations commonly encountered in the study setting. Response options were expanded to capture providers and places of death outside the formal health system, such as traditional birth attendants, religious settings, private clinics, public hospitals, and on the road. These changes helped reflect actual care-seeking and mortality contexts.
5. *Application of skip logic to streamline interview* To enhance efficiency, we digitally configured the tool to facilitate seamless navigation, allowing sections irrelevant to maternal deaths to be bypassed based on respondents’ answers. Respondents were first asked whether the deceased had certain conditions; only affirmative responses triggered follow-up questions. This digital configuration, which introduced skip logic, reduced the length of the interview similar to findings from other VA tools where digitisation and skip patterns reduced question burden by 40–55%, with minimal loss in diagnostic accuracy [15]. By streamlining navigation, we reduced respondent fatigue and improved operational feasibility in routine maternal death surveillance. Details of the skip logic applied are shown in Additional file 2.
6. *Data collection oversight features* The questionnaire was updated to include fields such as local government area, enumeration code, mapping ID, and Global Positioning System coordinates to enable precise identification of data collection sites. These additions supported effective monitoring, supervision, and quality assurance in line with the project protocol, enhancing data integrity and traceability.

A summary of the modifications made is presented in Table 2 and a copy of the adapted 2022 WHO VA tool can be found in Additional file 1.

**Table 2:** Summary of some key modifications carried out during the adaptation process

Change type	Example of modification	Reason/Benefit
Cultural appropriateness	Replaced “Ambiguous/Intersex” with “Female” or “Male” only	Aligns with local cultural sensitivities and avoids confusion among respondents.
Respect/Courtesy	Changed “What is your relationship to the deceased?” to “What is your relationship to her?”	Demonstrates empathy and respect for the deceased and their family.
Improved comprehension	“Abdominal pain” supplemented with “(tummy) pain”	Uses a less technical term to ensure better understanding among respondents without medical background.
Location-specific Options	Expanded the “Place of death” options to include “Religious house,” “Maternity home,” etc.	Reflects real-world circumstances under which maternal deaths can occur in the Lagos context.
Quality assurance	Added a statement: “I will be asking your permission to record…”	Ensures ethical practice, fosters trust with respondents and improves data recording accuracy.
Skip patterns	Re-ordered questions on communicable diseases vs. non-communicable diseases	Addresses potential stigma by starting with less sensitive health conditions before progressing to more stigmatized ones.
	Implemented conditional branching where questions about specific symptoms only appear if respondent indicates the deceased experienced those symptoms.	Reduces interview time, minimizes respondent burden, and ensures only relevant questions are asked based on previous responses.
Technical feature	Integrated GPS coordinates capture for precise location of the site of documentation of maternal death.	Enables accurate geographic mapping of maternal deaths for spatial analysis to inform policy; while maintaining respondent privacy.

#### Language translation

Language adaptation was essential for effective communication as many in the study population, especially in informal settlements and peri-urban areas, primarily speak Yoruba or Pidgin English (an English-based creole). Standard Yoruba was used, as it is widely understood across all Yoruba dialects in Lagos. Following the symmetrical translation framework proposed by Sousa et al., [9] the modified 2022 WHO VA tool was translated using a multi-step process: forward translation by two translators fluent in health terminology and local linguistic nuances, expert bilingual review by a medical doctor to identify discrepancies, independent back-translation into English by two different translators, and a final reconciliation meeting to resolve inconsistencies.

Linguistic validation was undertaken through multiple rounds of testing in diverse communities, including Yoruba-speaking households, non-Yoruba-speaking communities, and among final-year medical students serving as mock respondents. These steps confirmed that the adapted tool was comprehensible, culturally appropriate, and free of significant semantic loss, ensuring fidelity to the original meaning while enhancing local resonance.

#### Validation and piloting of the adapted tool

The adapted tool underwent a rigorous multi-stage validation process. Face validity was assessed by non-experts: final year medical students, data collectors, and data analysts to assess its clarity, readability, comprehension, and appropriateness [16]. A team of experts from Obstetrics and Gynaecology, Public Health, Maternal Health, Epidemiology, and Statistics conducted content validation to ensure completeness, relevance, simplicity, and alignment with WHO standards. During this phase, the tool was also tested for compatibility with the InterVA-5 automated VA coding algorithm, which was in use while awaiting the WHO’s integration of the 2022 VA standard. Outputs were reviewed by the project’s technical team, including epidemiologists and maternal health specialists, to confirm plausibility of assigned causes of death within the study context; no incompatibilities were identified, and the adapted tool retained full functionality. Feedback from expert group review meetings informed refinements to improve quality and contextual relevance.

Following the validation of the tool, Simulation interviews with trained data collectors and mock respondents tested flow, skip patterns, and functionality, allowing refinements. Pilot interviews were conducted with respondents from diverse age groups, educational backgrounds, religions, and socioeconomic contexts, and feedback confirmed that the adapted tool was understandable and culturally appropriate across these demographic subgroups, with no major comprehension issues specific to any group. Finally, the tool was piloted in two additional households affected by maternal deaths, where feedback from both interviewers and bereaved families confirmed improved engagement, cultural appropriateness, and data quality, guiding final adjustments before field deployment.

### Interviewer training

Before deploying the adapted tool in the field, data collectors underwent a comprehensive training programme grounded in the WHO VA training manual. The training aimed to ensure a thorough understanding of the tool’s structure, including its questions, response options, skip patterns, narrative text fields, and ethical standards. Emphasis was placed on maternal death sensitivity, with sessions on consent, psychological handling, and communication with bereaved families. Interview simulations were conducted to familiarise data collectors with real-life scenarios, highlight potential challenges, and reinforce appropriate responses, including when to pause interviews or initiate referrals for psychological support. Data collectors were also trained to manage their own emotional well-being and were provided access to mental health support services to mitigate the psychological toll of conducting such emotionally charged interviews.

### Implication for future research and policy

Tailoring global health instruments to local epidemiological, linguistic, and health system contexts is good practice. Adapting the 2022 WHO Verbal Autopsy tool in Lagos was essential to ensure it was fit for purpose. The adaptation went beyond language and cultural tailoring to include technical adjustments (e.g., skip logic), integration with local health system realities (e.g., care providers, place of death), and interviewer training that addressed the emotional complexity of engaging with bereaved families. By documenting this multi-level adaptation process, we provide a replicable model for other low-resource, multicultural settings seeking to strengthen mortality surveillance systems. Building on this, at the policy level, the adapted verbal autopsy tool is being integrated into the Lagos community-based MPDSR system, creating an opportunity to institutionalise its use in routine maternal mortality surveillance beyond the project. Embedding the tool in MPDSR processes can support systematic identification of maternal deaths occurring outside facilities, strengthen linkages between community and facility reporting, and ensure that lessons from each review inform preventive actions at scale.

## Strengths and limitations

This paper provides a methodologically transparent and context-sensitive adaptation of the 2022 WHO VA tool for maternal mortality surveillance in Lagos State, in line with the WHO guidelines and established frameworks. Its alignment with WHO guidelines and adaptation to local health system constraints enhances its potential utility in similar low-resource settings. However, it does not evaluate the tool’s diagnostic accuracy or inter-rater reliability in this setting. Additionally, the adaptation focused primarily on maternal deaths, and findings may not be generalisable to other population groups. Future studies could assess the tool’s performance and longitudinal usability across other verbal autopsy modules.

## Supplementary Information

Below is the link to the electronic supplementary material.

Supplementary Material 1.

Supplementary Material 2.

## Data Availability

The adapted verbal autopsy tool is included as an additional file. No extra data were generated or analysed during the current study.

## Abbreviations

CoD: Cause of Death
CRVS: Civil Registration and Vital Statistics
LMICs: Low- and Middle-Income Countries
LVASA-SRS: Lagos State Verbal Autopsy and Social Autopsy Sample Registration System
MPDSR: Maternal and Perinatal Death Surveillance and Response
VA: Verbal Autopsy
WHO: World Health Organization
