# Supplementary material for: Adapting the 2022 WHO verbal autopsy tool for use in Lagos State, Nigeria: insights from the LVASA-SRS project
Source: BMC Res Notes. 2025 Dec 29;18:508. doi: 10.1186/s13104-025-07560-1 (PMC12751495; doi:10.1186/s13104-025-07560-1)
Supplement: Supplementary file 1 — Supplementary Material 1. [file 13104_2025_7560_MOESM1_ESM.docx]

ADDITIONAL FILE 1: VASA INTERVIEW

# Information of the interviewer

Id10010 - Name of VA interviewer

Id10010a - Age of VA interviewer

Id10010b - Sex of VA interviewer


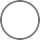
 Female
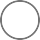
 Male

Id10010c - ID of VA interviewer

Interview language


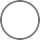
 English
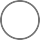
 Yoruba
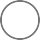
 Pidgin English

# Information on the respondent and background about interview

* Thank you for considering participating in our survey on maternal mortality and stillbirth. The purpose of this survey is to gather information that can help us understand the causes of maternal deaths and stillbirths in our community. Your participation is voluntary, and you have the right to refuse to participate or withdraw from the survey at any time without any consequences. As part of the survey, we will be conducting verbal autopsies. A verbal autopsy is a method used to determine the probable cause of death by interviewing family members or caregivers of the deceased. The information collected during the verbal autopsy will be used for research purposes only and will be kept confidential. Your identity and the identity of the deceased will not be disclosed in any reports or publications. Participation in the survey involves answering questions about the circumstances leading to maternal death or stillbirth. The questions may be sensitive, so please feel free to skip any questions that you do not wish to answer. If you have any questions or concerns about the survey or the verbal autopsy process, please contact Mr. Ndubuisi Ezumezu, Centre for Clinical Trials, Research and Implementation Science, College of Medicine, University of Lagos; +2348063754883. By providing your consent, you agree to participate in the survey and authorize us to conduct a verbal autopsy to gather information about the maternal death or stillbirth. Your participation is valuable and will help us improve maternal and child health in our community. Do you have any questions or concerns? If not, could you please provide your verbal consent to participate in the survey and the verbal autopsy process?


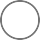
OK

Respondent Signature

Respondent Thumbprint Consent

Click here to upload file. (< 10MB)

Respondent Initials

*If the respondent cannot sign, please enter the initials of the names*

I will be asking your permission to record. The reason is, I want to spend time listening to you, rather than taking notes. So, we can go back and capture the details. Stop the recording above if the respondent disagrees.


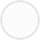
 Agree
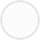
 Disagree

Id10007 - What is the full name of VA respondent?

Id10007a - What is the sex of VA respondent?


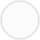
 Female
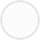
 Male

Id10007b - What is the age of VA respondent?


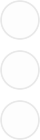

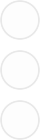
Id10008 - What is your relationship with her?


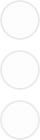


| Parent | Child | Other family member |
| --- | --- | --- |
| Friend | Spouse | Health worker |
| Public official | Another relationship | Refused to answer |

Id10009 - Did you live with her in the period leading to her death?


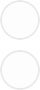
Yes
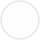
 No
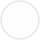
 Doesn't Know Refused to answer

Id10012 - Date of the interview

# House Locator Details - To be completed by VA Interviewer


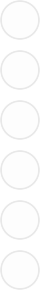

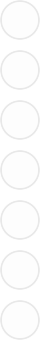
Local Government Area


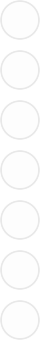


| Agege | Ajeromi-Ifelodun | Alimosho |
| --- | --- | --- |
| Amuwo odofin | Apapa | Badagry |
| Epe | Eti-Osa | Ibeju-Lekki |
| Ifako-Ijaiye | Ikeja | Ikorodu |
| Kosofe | Lagos Island | Lagos Mainland |
| Mushin | Ojo | Oshodi-Isolo |
| Somolu | Surulere |  |

Enumeration Area Code

Mapping ID

Geolocation

latitude (x.y °)


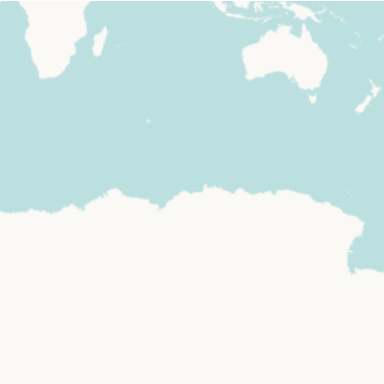


longitude (x.y °)

altitude (m)

accuracy (m)

# Information about the deceased and vital registration - Information on the Deceased

Id10017- What was her first or given names?

Id10018 - What was her surnames or family names?

Id10019 - What was the sex of the deceased?


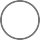
 Female
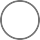
 Male

Id10020 - Is the date of birth known?


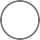
 Yes
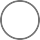
 No


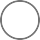
 Refused to answer

Id10021 - When was she born?

Age in years

Id10022 - Is the date of death known?


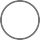
 Yes
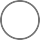
 No


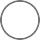
 Refused to answer

Id10023_a - When did she die?

Id10024 - Please indicate the year of death.


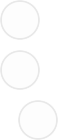

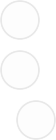
Id10058 - Where did she die?


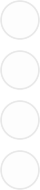


| Home (place of residence) | Private hospital | Public hospital |
| --- | --- | --- |
| Maternity home | Traditional birth attendant | Religious house |
| On route to hospital or facility | Other Please specify | Doesn't know |
| Refused to answer |  |  |

Please specify other

Id10487 - In the two weeks before death, did she live with, visit, or care for someone who had any COVID-19 symptoms, or a positive COVID-19 test?

*COVID-19 symptoms include fever, difficulty breathing, cough, extreme fatigue, and changes in sense of smell or taste. In case of neonates or young children, please omit "care for".*


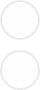
Yes
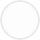
 No
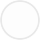
 Doesn't know Refused to answer

Id10052 - What was her citizenship/nationality?


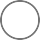
 Nigerian


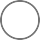
 Non-Nigerian
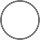
 Doesn't know


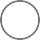
 Refused to answer

Id10053 - What was her ethnicity?


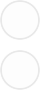
Hausa
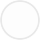
 Igbo
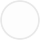
 Yoruba Others

Other Ethnicity

Id10054 - What was her place of birth?

*Specify here town/city and state. A question on the facility and circumstances will be asked later. Enter a "-" if this information is not available.*

Id10055 - What was her place of usual residence? the place where the person lived most of the year

*The home address.*

Id10057 - Where did the death occur? specify town/city, State

Id10059 - What was her marital status?

*
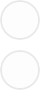

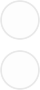
Life partner is defined here as living with someone for a long time without ever having married.*

*
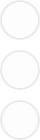
*

| Single | Married | Cohabiting |
| --- | --- | --- |
| Divorced | Widowed | Doesn't know |
| Refused to answer |  |  |

Id10063 - What was her highest education attained?


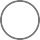
 No formal education
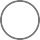
 Primary school


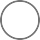
 Secondary school


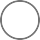
 Higher than secondary school
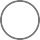
 Doesn't know


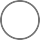
 Refused to answer

Id10064 - Was she able to read and/or write?


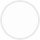
 Yes
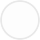
 No


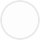
 Doesn't know


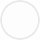
 Refused to answer

Id10065 - What was her economic activity status in year prior to death?

*
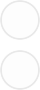

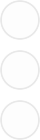
The deceased might have had several activities. Choose the one that was probably true for most of the year preceding illness and death*

*
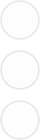
*

| Unemployed | Employed | Housewife |
| --- | --- | --- |
| Pensioner | Student | Other |
| Doesn't know | Refused to answer |  |

Open narrative - If needed, probe the respondent for additional details on

when the deceased recognized symptoms, abnormalities, care sought, etc. Ask the respondent if any medical records from the time preceding death are available and record any relevant information. Some of the following questions may be repetitive or irrelevant to the respondent but they are very important

in the COD assignment process.

Id10476 - Thank you for your information. Now can you please tell me in your own words about the events that led to the death?

Id10477 - Select any of the following words that were mentioned as present in the narrative

*Choose one or more options:
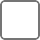
* chronic kidney disease
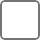
 Dialysis


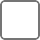
 Renal kidney failure


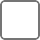
 Heart attack
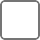
 Heart problem
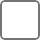
 Jaundice


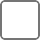
 Liver failure
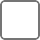
 Malaria


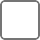
 Pneumonia
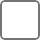
 Fever


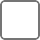
 Suicide


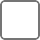
 None of the above words were mentioned
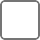
 Don't know

# Medical history associated with final illness. Explain to the respondent that the following section contains a series of questions on whether diagnosis from a

health professional was obtained for a number of illnesses. Clarify that the aim of this series is on medical diagnosis of specific illnesses, and not on signs and symptoms or the perceived cause of death by the respondent.

Id10128 - Did she have a recent positive test by a health professional for malaria?

*Remind the respondent that we are asking for the diagnosis assessed by a doctor, health worker, or other health professional during the final illness.*


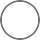
 Yes
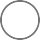
 No


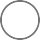
 Doesn't know


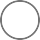
 Refused to answer

Id10129 - Did she have a recent negative test by a health professional for malaria?

*Remind the respondent that we are asking for the diagnosis assessed by a doctor, health worker, or other health professional during the final illness.*


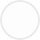
 Yes
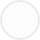
 No


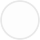
 Doesn't know


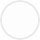
Refused to answer

Id10131 - Was there any diagnosis by a health professional of measles?

*Remind the respondent that we are asking for the diagnosis assessed by a doctor, health worker, or other health professional during the final illness.*


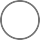
 Yes
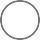
 No


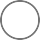
 Doesn't know


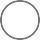
 Refused to answer

Id10132 - Was there any diagnosis by a health professional of high blood pressure?

*Remind the respondent that we are asking for the diagnosis assessed by a doctor, health worker, or other health professional during the final illness.*


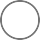
 Yes
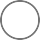
 No


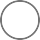
 Doesn't know


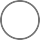
 Refused to answer

Id10135 - Was there any diagnosis by a health professional of asthma?

*Remind the respondent that we are asking for the diagnosis assessed by a doctor, health worker, or other health professional during the final illness.*


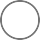
 Yes
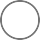
 No


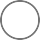
 Doesn't know


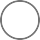
 Refused to answer

Id10134 - Was there any diagnosis by a health professional of diabetes?

*Remind the respondent that we are asking for the diagnosis assessed by a doctor, health worker, or other health professional during the final illness.*


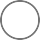
 Yes
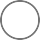
 No


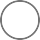
 Doesn't know


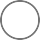
 Refused to answer

Id10136 - Was there any diagnosis by a health professional of epilepsy?

*Remind the respondent that we are asking for the diagnosis assessed by a doctor, health worker, or other health professional during the final illness.*


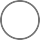
 Yes
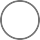
 No


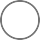
 Doesn't know


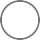
Refused to answer

Id10139 - Was there any diagnosis by a health professional of dementia?

*Remind the respondent that we are asking for the diagnosis assessed by a doctor, health worker, or other health professional during the final illness.*


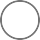
 Yes
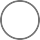
 No

Doesn't know

Refused to answer

Id10140 - Was there any diagnosis by a health professional of depression?

*Remind the respondent that we are asking for the diagnosis assessed by a doctor, health worker, or other health professional during the final illness.*

Yes No

Doesn't know

Refused to answer

Id10141 - Was there any diagnosis by a health professional of stroke?

*Remind the respondent that we are asking for the diagnosis assessed by a doctor, health worker, or other health professional during the final illness.*

Yes No

Doesn't know

Refused to answer

Id10133 - Was there any diagnosis by a health professional of heart disease?

*Remind the respondent that we are asking for the diagnosis assessed by a doctor, health worker, or other health professional during the final illness.*

Yes No

Doesn't know

Refused to answer

Id10130 - Was there any diagnosis by a health professional of Lassa fever?

*Remind the respondent that we are asking for the diagnosis assessed by a doctor, health worker, or other health professional during the final illness.*

Yes No

Doesn't know

Refused to answer

Id10142 - Was there any diagnosis by a health professional of sickle cell disease?

*Remind the respondent that we are asking for the diagnosis assessed by a doctor, health worker, or other health professional during the final illness.*

Yes No

Doesn't know

Refused to answer

Id10138 - Was there any diagnosis by a health professional of Chronic Obstructive Pulmonary Disease COPD?

*Remind the respondent that we are asking for the diagnosis assessed by a doctor, health worker, or other health professional during the final illness.*

Yes No

Doesn't know

Refused to answer

Id10143 - Was there any diagnosis by a health professional of kidney disease?

*Remind the respondent that we are asking for the diagnosis assessed by a doctor, health worker, or other health professional during the final illness.*

Yes No

Doesn't know

Refused to answer

Id10144 - Was there any diagnosis by a health professional of liver disease?

*Remind the respondent that we are asking for the diagnosis assessed by a doctor, health worker, or other health professional during the final illness.*

Yes No

Doesn't know

Refused to answer

Id10125 - Was there any diagnosis by a health professional of tuberculosis?

*Remind the respondent that we are asking for the diagnosis assessed by a doctor, health worker, or other health professional during the final illness.*

Yes No

Doesn't know

Refused to answer

Id10482 - Was there any diagnosis by a health professional of COVID-19?

*Remind the respondent that we are asking for the diagnosis assessed by a doctor, health worker, or other health professional during the final illness.*

Yes No

Doesn't know

Refused to answer

Id10483 - Did she have a recent test for COVID-19?

*Remind the respondent that we are asking for the diagnosis assessed by a doctor, health worker, or other health professional during the final illness.*

Yes No

Doesn't know

Refused to answer

Id10484 - What was the result?

*Prompt for the result of the most recent test in case the deceased had more than 1 test performed*

Positive Negative Unclear Don't know

Refused to answer

Id10126 - Was an HIV test ever positive?

*Remind the respondent that we are asking for the diagnosis assessed by a doctor, health worker, or other health professional during the final illness.*

Yes No

Doesn't know

Refused to answer

Id10127 - Was there any diagnosis by a health professional of AIDS?

*Remind the respondent that we are asking for the diagnosis assessed by a doctor, health worker, or other health professional during the final illness.*

Yes No

Doesn't know

Refused to answer

Id10137 - Was there any diagnosis by a health professional of cancer?

*Remind the respondent that we are asking for the diagnosis assessed by a doctor, health worker, or other health professional during the final illness.*

Yes No

Doesn't know

Refused to answer

# Unless specified, the following questions on signs, symptoms, treatment and

circumstances relate specifically to the illness and the period of illness that led to death. History of injuries/accidents.

Id10077 - Did she suffer from any injury or accident that led to her death?

*Remind the respondent that we are asking for the diagnosis assessed by a doctor, health worker, or other health professional during the final illness.*

Yes No

Doesn't know

Refused to answer

# Injuries and accidents detail

Id10077_a - How long after the injury or accident did, she die?

Less or equal to 7 days More than 7 days

Don't know

Refused to answer

* Id10077_b - Click to confirm the answer: She/died less than or equal to 7 days after the accident OK

Id10079 - Was it a road traffic injury?

Yes No

Doesn't know

Refused to answer

Id10082 - Was it a non-road traffic injury?

*Non-road traffic injuries include those involving air e.g., plane, rail e.g., train, sea or river e.g. Boat, canoe transportation*

Yes No

Doesn't know

Refused to answer

Id10083 - Was she injured in a fall?

*This includes accidents and cases where it is unknown if it was an accident or whether there was intentional violence.*

Yes No

Doesn't know

Refused to answer

Id10084 - Was there any poisoning?

*This includes accidents and cases where it is unknown if it was an accident or whether there was intentional violence.*

Yes No

Doesn't know

Refused to answer

Id10085 - Did she die of drowning?

*This includes accidents and cases where it is unknown if it was an accident or whether there was intentional violence.*

Yes No

Doesn't know

Refused to answer

Id10086 - Was she injured by a venomous bite or sting from an animal or insect?

*This includes accidents and cases where it is unknown if it was an accident or whether there was intentional violence.*

Yes No

Doesn't know

Refused to answer

Id10087 - Was she injured by an animal or insect non-venomous?

Yes No

Doesn't know

Refused to answer

Id10088 - What was the animal/insect?

Dog Snake

Insect or scorpion Others Please specify Doesn't know

Refused to answer

Others Specify

Id10089 - Was she injured by burns/fire?

Yes No

Doesn't know

Refused to answer

Id10091 - Was she injured by a firearm?

Yes No

Doesn't know

Refused to answer

Id10092 - Was she stabbed, cut or pierced?

Yes No

Doesn't know

Refused to answer

Id10093 - Was she strangled?

Yes No

Doesn't know

Refused to answer

Id10096 - Was she electrocuted?

Yes No

Doesn't know

Refused to answer

Id10094 - Was she injured by a blunt force?

*A blunt force trauma is a non-penetrating injury from an object.*

Yes No

Doesn't know

Refused to answer

Id10095 - Was she injured by a force of nature?

*Forces of nature can include lightning, flooding, earthquake, tsunami, bush fire, volcanic eruption, etc.*

Yes No

Doesn't know

Refused to answer

Id10097 - Did she suffer any other injury?

Yes No

Doesn't know

Refused to answer

Id10098 - Was the injury accidental?

Yes No

Doesn't know

Refused to answer

Id10099 - Was the injury self-inflicted?

Yes No

Doesn't know

Refused to answer

Id10100 - Was the injury intentionally inflicted by someone else?

Yes No

Doesn't know

Refused to answer

# Health history - Duration of illness

id10120_unit - For how long was she ill before death?

Days - less than 24 hours = 0 days Months

Years

Doesn't know

Refused to answer

Id10121 - Months

Id10122 - Years

Id10120_1 - Days

*Less than 24 hours = 0 days.*

Id10123 - Did she die suddenly?

Yes No

Doesn't know

Refused to answer

# General signs and symptoms associated with final illness

Id10147 - Did she have a fever?

*Fever is a term used when the body feels abnormally warm or hot to touch, and/or when a thermometer records an abnormally high temperature.*

Yes No

Doesn't know

Refused to answer

Id10148_units - How long did the fever last?

*If the respondent is unable to answer, prompt: Did the fever last less than 1 week interviewer to enter 6 days; less than two weeks interviewer to enter 13 days; or more than 2 weeks interviewer to enter 15 days? Enter 1 unit only: 0-30days or 1-60 months. Less than 1 day or 24 hours = 0 days; 1 week = 7 days.*

Days Months

Doesn't know

Refused to answer

Id10148_b - Enter how long the fever lasted in days:

*Enter 0-30 days. Less than 1 day or 24 hours = 0 days; 1 week = 7 days.*

Id10148_c - Enter how long the fever lasted in months

*Enter 1-60 months.*

Id10149 - Did the fever continue until death?

Yes No

Doesn't know

Refused to answer

Id10150 - How severe was the fever?

Mild Severe

Doesn't know

Refused to answer

Id10151 - What was the pattern of the fever?

Continuous On and off Only at night Doesn't know

Refused to answer

Id10153 - Did she have a cough?

Yes No

Doesn't know

Refused to answer

Id10154_units - For how long did she have a cough?

*If the respondent is unable to answer, prompt: Did the cough last less than 3 weeks interviewer to enter 20 days; or at least 3 weeks interviewer to enter 22 days? Enter 1 unit only: 0-30 days or 1-60 months. Less than 1 day or 24 hours = 0days; 1 week = 7 days.*

Days Week Months

Doesn't know

Refused to answer

Id10154_a - Enter how long she had a cough in days:

*Enter 0-30 days. Less than 1 day or 24 hours = 0 days; 1 week = 7 days.*

Id10154_b - Enter how long she had a cough in months:

*Enter 1-60 months.*

Id10155 - Was the cough productive, with sputum?

Yes No

Doesn't know

Refused to answer

Id10156 - Was the cough very severe?

Yes No

Doesn't know

Refused to answer

Id10157 - Did she cough up blood?

Yes No

Doesn't know

Refused to answer

Id10159 - Did she have any difficulty breathing or breathlessness?

*Breathing difficulties are an important feature that aid identification of the cause of death and can be observed in the form of struggling to breath or feeling out of breath.*

Yes No

Doesn't know

Refused to answer

# Duration of breathing difficulty

Id10161_unit - For how long did the difficulty breathing or breathlessness last?

*If the respondent is unable to answer, prompt: Did the difficulty breathing or breathlessness last for less than 3 days interviewer to enter 2 days, or for at least 3 days interviewer to enter 4 days?*

Days Week Months

Doesn't know

Refused to answer

Id10161_1 - Enter how long the difficult breathing or breathlessness lasted in days:

*Enter 0-30 days. Less than 1 day or 24 hours = 0 days; 1 week = 7 days.*

Id10162 - Enter how long the difficult breathing or breathlessness lasted in months:

*Enter 1-60 months.*

Id10163 - Enter how long the difficult breathing or breathlessness lasted in years:

*Enter number of years less than age at death.*

Id10165 - Was the difficulty in breathing continuous or on and off?

Continuous On and off Doesn't know

Refused to answer

Id10170 - Was she unable to carry out daily routines due to breathlessness?

*A person with severe breathlessness will struggle to do daily routines, such as walking short distance or taking a bath, and will need assistance from someone.*

Yes No

Doesn't know

Refused to answer

Id10171 - Was she breathless while lying flat?

Yes No

Doesn't know

Refused to answer

Id10166 - Did she have fast breathing?

Yes No

Doesn't know

Refused to answer

Id10167_units - How long did the fast breathing last?

*If the respondent is unable to answer, prompt: Did the difficulty breathing last for less than 2 weeks interviewer to enter13 days, or for at least 2 weeks interviewer to enter 15 days?*

Days Week Months

Doesn't know

Refused to answer

Id10167_b - Enter how long the fast breathing lasted in days:

*Enter 0-30 days. Less than 1 day or 24 hours = 0 days; 1 week = 7 days.*

Id10167_c - Enter how long the fast breathing lasted in months:

*Enter 1-60 months.*

Id10173_a - Did she have wheezing?

Yes No

Doesn't know

Refused to answer

Id10174 - Did she have chest pain?

Yes No

Doesn't know

Refused to answer

Id10175 - Was the chest pain severe?

Yes No

Doesn't know

Refused to answer

Id10176 - How many days before death did, she has chest pain?

*If the respondent is unable to answer, prompt: Did she have chest paint for less than 3 days before death interviewer to enter 2 days, or for at least more than 3 days before death interviewer to enter 4 days? Less than 1 day or 24 hours = 0days; 1 week = 7 days. For don't know, enter "99." For refused, enter "88."*

# Duration of the chest pain

Id10178_unit - How long did the chest pain last?

*Round up the response given by the respondent as needed e.g. if chest pain lasted for 2 hours 30 mins; enter 3 hours. If the respondent is unable to answer, prompt: Did the chest pain last for less than 1 hour interviewer to enter 0hours,1 to 4 hours interviewer to enter 4 hours, 5 to 23 hours interviewer to enter 23 hours.*

Days Week Months

Doesn't know

Refused to answer

Id10179 - Enter how long the chest pain lasted in hours:

*Enter 0-23 hours.*

Id10179_1 - Enter how long the chest pain lasted in days:

*Enter 0-30 days. 1 week = 7 days.*

# Group

Id10181 - Did she have diarrhoea?

*Ask the respondent about his/her understanding of what is diarrhoea having more frequent loose or liquid stools than usual; if unclear, explain to the respondent what diarrhoea is . Diarrhoea means having more frequent loose or liquid stools than usual.*

Yes No

Doesn't know

Refused to answer

Id10182_units - How long did she have diarrhoea?

*If the respondent is unable to answer, prompt: Did the diarrhoea last for less than 2 weeks interviewer to enter 13 days; between two to four weeks interviewer to enter 15 days; or for more than 4 weeks interviewer to enter 29 days?*

Days Week Months

Doesn't know

Refused to answer

Id10182_a - Enter how long she have diarrhoea in days:

*Enter 0-30 days. Less than 1 day or 24 hours = 0 days; 1 week = 7 days.*

Id10182_b - Enter how long she have diarrhoea in months:

*Enter 1-60 months.*

Id10186 - At any time during the final illness, was there blood in the stools?

Yes No

Doesn't know

Refused to answer

Id10188 - Did she vomit?

Yes No

Doesn't know

Refused to answer

Id10190_units - For how long did she vomit?

*If the respondent is unable to answer, prompt: Did she vomit for less than 3 days interviewer to enter 2 days, or for more than 3 days interviewer to enter 4 days? Enter 1 unit only: 0-30 days or 1-60 months. Less than 1 day or 24 hours= 0 days; 1 week = 7 days.*

Days Week Months

Doesn't know

Refused to answer

Id10190_c - Enter how long before death she vomited in hours:

Id10190_a - Enter how long before death she vomited in days:

Id10190_b - Enter how long before death she vomited in months:

Id10189 - Did she vomit in the week preceding the death?

Yes No

Doesn't know

Refused to answer

Id10189_1 - Did she vomit every time she ate and/or drank?

Yes No

Doesn't know

Refused to answer

Id10192 - Was the vomit black?

Yes No

Doesn't know

Refused to answer

Id10194 - Did she have abdominal (tummy) pain ?

Yes No

Doesn't know

Refused to answer

Id10195 - Was the abdominal (tummy) pain severe?

Yes No

Doesn't know

Refused to answer

# Abdominal pain

id10196_unit - For how long did she have abdominal (tummy) pain?

*If the respondent is unable to answer, prompt: Did the abdominal (tummy) pain last for less than 2 weeks interviewer to enter 13days, or for at least 2 weeks interviewer to enter 15 days? Enter 1 unit only: 0-23 hours, 1-30 days, or 1-60 months. 1week = 7 days.*

Days Week Months

Doesn't know

Refused to answer

Id10196 - Enter how long she had abdominal (tummy) pain in hours:

Id10197_a - Enter how long she had abdominal (tummy) pain in days:

*Enter 0-30 days. 1 week = 7 days.*

Id10198 - Enter how long she had abdominal (tummy) pain in months:

*Enter 1-60 months.*

Id10199 - Where was the location of the abdominal (tummy) pain?

Upper right abdomen Upper left abdomen Lower right abdomen Lower left abdomen Mid abdomen

All over the abdomen Doesn't know

Refused to answer

Id10200 - Did she have a more than usually protruding abdomen (tummy)?

*A more than usual protruding abdomen presents as an expansion of the whole abdomen. A protruding abdomen is different from an abdominal mass that shows as a localised enlargement in the abdomen.*

Yes No

Doesn't know

Refused to answer

Id10201_unit - For how long before death did she have a more than usually protruding abdomen (tummy)?

*If the respondent is unable to answer, prompt: Did she have a more than usual protruding abdomen for less than 2 weeks interviewer to enter 13 days, or for at least 2 weeks interviewer to enter 15 days? Enter 1 unit only: 0-30 days or 1-60months. Less than 1 day or 24 hours*

*= 0 days; 1 week = 7 days.*

Days Week Months

Doesn't know

Refused to answer

Id10201_a - Enter how long before death she had a more than usually protruding abdomen (tummy) in days:

Id10202 - Enter how long before death she had a more than usually protruding abdomen (tummy) in months:

Id10203 - How rapidly did she develop the protruding abdomen (tummy)?

Rapidly Slowly

Doesn't know

Refused to answer

Id10204 - Did she have any mass in the abdomen (tummy)?

Yes No

Doesn't know

Refused to answer

Id10205_unit - For how long did she have a mass in the abdomen (tummy)?

*If the respondent is unable to answer, prompt: Did she have a mass in the abdomen for less than 2 weeks interviewer enter 13 days, or for at least 2 weeks interviewer to enter 15 days? Enter 1 unit only: 0-30 days or 1-60 months. Less than 1 day or 24 hours = 0 days; 1 week = 7 days.*

Days Week Months

Doesn't know

Refused to answer

Id10205_a - Enter how long she had a mass in the abdomen (tummy) in days

*Enter 0-30 days. Less than 1 day or 24 hours = 0 days; 1 week = 7 days.*

Id10206 - Enter how long she had a mass in the abdomen (tummy) in months:

*Enter 1-60 months.*

Id10207 - Did she have a severe headache?

Yes No

Doesn't know

Refused to answer

Id10208 - Did she have a stiff or painful neck?

*Choose only one option:*

Yes No

Doesn't know

Refused to answer

Id10209_units - How long before death did she have a stiff or painful neck?

*If the respondent is unable to answer, prompt: Did she have a stiff or painful neck for less than 1 week interviewer to enter 6 days, or for at least 1 week interviewer to enter 8 days? Enter 1 unit only: 0-30 days or 1-60 months. Less than 1day or 24 hours = 0 days; 1 week = 7 days.*

Days Week Months

Doesn't know

Refused to answer

Id10209_a - Enter how long before death did she have stiff or painful neck in days:

*Enter 0-30 days. Less than 1 day or 24 hours = 0 days; 1 week = 7 days.*

Id10209_b - Enter how long before death did she have stiff or painful neck in months:

*Enter 1-60 months.*

Id10212 - Did she have mental confusion?

*Choose only one option:*

Yes No

Doesn't know

Refused to answer

Id10213_units - How long did she have mental confusion?

*If the respondent is unable to answer, prompt: Did the mental confusion last for less than 3 months interviewer to enter 2months, or for at least 3 months interviewer to enter 4 months? Enter 1 unit only: 0-30 days or 1-60 months. Less than 1day or 24 hours = 0 days; 1 week = 7 days.*

Days Week Months

Doesn't know

Refused to answer

Id10213_a - Enter how long she had mental confusion in days:

*Enter 0-30 days. Less than 1 day or 24 hours = 0 days; 1 week = 7 days.*

Id10213_b - Enter how long she had mental confusion in months:

*Enter 1-60 months.*

Id10214 - Was she unconscious?

*Choose only one option:*

Yes No

Doesn't know

Refused to answer

Id10216_units - How long before death did unconsciousness start?

*If the respondent is unable to answer, prompt: Did the unconsciousness start less than 6 hours before death interviewer to enter 5 hours, did it start between 6- and 23-hours interviewer to enter 23 hours; or did it start at least 24 hours before death interviewer to enter 1 day? Enter 1 unit only: 0-23 hours or 1-99 days. 1 week = 7 days.*

Days Week Months

Doesn't know

Refused to answer

Id10216_a - Enter how long before death unconsciousness started in hours?

*The question needs input in hours but the respondent may not know exactly and so it may be easier to ask, 'how long' and then convert the duration in hours. Less than 1 hour = "0".*

Id10216_b - Enter how long before death unconsciousness started in days?

*If more than 99, enter 99.*

Id10217 - Did the unconsciousness start suddenly, quickly at least within a single day?

Yes No

Doesn't know

Refused to answer

Id10220 - Did she experience any generalized convulsions?

*Convulsions are rapid twitching or jerking movements of the whole body i.e. both arms and both legs, which frequently subside with the loss of consciousness. The common term for convulsions is fits, and there is often a local term for such movements.*

Yes No

Doesn't know

Refused to answer

Id10222 - Did she become unconscious immediately after the convulsion?

Yes No

Doesn't know

Refused to answer

Id10223 - Did she have any urine problems?

*Urine problems can include pain or difficulty in passing urine, passing blood in the urine or unable to urinate.*

Yes No

Doesn't know

Refused to answer

Id10226 - During the final illness did she ever pass blood in the urine?

Yes No

Doesn't know

Refused to answer

Id10224 - Did she stop urinating?

*This means that the deceased stopped urinating and did not urinate again in the 24 hours or more before death.*

Yes No

Doesn't know

Refused to answer

Id10230 - Did she have an ulcer (wound) on the foot?

Yes No

Doesn't know

Refused to answer

Id10231 - Did the ulcer (wound) on the foot have pus?

Yes No

Doesn't know

Refused to answer

Id10232_units - How long did the ulcer (wound) on the foot have pus?

*If the respondent is unable to answer, prompt: Did the ulcer on the foot have pus for less than 2 weeks interviewer to enter13 days, or for at least 2 weeks interviewer to enter 15 days?*

Days Week Months

Doesn't know

Refused to answer

Id10232_a - Enter how long the ulcer (wound) on the foot had pus in days:

*Enter 0-30 days. Less than 1 day or 24 hours = 0 days; 1 week = 7 days.*

Id10232_b - Enter how long the ulcer (wound) on the foot had pus in months:

*Enter 1-60 months.*

Id10227 - Did she have ulcers (wounds) or sores anywhere else on the body?

*ulcers and sores refer to breaking of the skin or mucous membranes that are slow to heal or keep returning.*

Yes No

Doesn't know

Refused to answer

Id10229 - Did the ulcers (wounds) or sores have pus?

Yes No

Doesn't know

Refused to answer

Id10233 - Did she have any skin rash?

Yes No

Doesn't know

Refused to answer

Id10234 - For how many days did she have the skin rash?

*If the respondent is unable to answer, prompt: Did the skin rash last for less than 1 week interviewer to enter 6 days, or for at least 1 week interviewer to enter 8 days? . Less than 1 day or 24 hours = 0 days; 1 week=7 days; 1 month=30days. Enter "99" for "don't know." Enter "88" for "refuse."*

Id10235 - Where was the rash?

Face

Trunk or abdomen (tummy) Extremities (hands and legs) Everywhere

Doesn't know

Refused to answer

Id10236 - Did she have measles rash?

Yes No

Doesn't know

Refused to answer

Id10237 - Did she ever have shingles (painful rash) or herpes zoster (chicken pox)?

Yes No

Doesn't know

Refused to answer

Id10238 - Did her skin flake off (peel off) in patches?

Yes No

Doesn't know

Refused to answer

Id10242 - Did she bleed from the nose, eyes, mouth or anus?

Yes No

Doesn't know

Refused to answer

Id10243 - Did she have noticeable weight loss?

Yes No

Doesn't know

Refused to answer

Id10244 - Was she severely thin or wasted?

Yes No

Doesn't know

Refused to answer

Id10245 - Did she have a whitish rash inside the mouth or on the tongue?

Yes No

Doesn't know

Refused to answer

Id10246 - Did she have stiffness of the whole body or was unable to open the mouth?

Yes No

Doesn't know

Refused to answer

Id10247 - Did she have swollen /puffiness of the face?

Yes No

Doesn't know

Refused to answer

Id10248_units - How long did she have swollen/ puffiness of the face?

*If the respondent is unable to answer, prompt: Did the swollen/ puffiness of the face last for less than 1 week interviewer to enter 6days, or for at least 1 week interviewer to enter 8 days? Enter 1 unit only: 0-30 days or 1-60 months. Less than 1-day or24 hours = 0 days; 1 week = 7 days.*

Days Week Months

Doesn't know

Refused to answer

Id10248_a - Enter how long she had swollen/puffiness of the face in days:

*Enter 0-30 days. Less than 1 day or 24 hours = 0 days; 1 week = 7 days.*

Id10248_b - Enter how long she had swollen/puffiness of the face in months:

*Enter 1-60 months.*

Id10249 - Did she have swollen legs or feet?

Yes No

Doesn't know

Refused to answer

Id10250_units - How long did the swelling last?

*If the respondent is unable to answer, prompt: Did the swelling last for less than 3 days interviewer to enter 2 days, or for more than 3 days interviewer to enter 4 days? Enter 1 unit only: 0-30 days or 1-60 months. Less than 1 day or 24 hours= 0 days; 1 week = 7 days.*

Days Week Months

Doesn't know

Refused to answer

Id10250_a - Enter how long the swelling lasted in days

*Enter 0-30 days. Less than 1 day or 24 hours = 0 days; 1 week = 7 days.*

Id10250_b - Enter how long the swelling lasted in months:

*Enter 1-60 months.*

Id10251 - Did she have both feet swollen?

Yes No

Doesn't know

Refused to answer

Id10252 - Did she have general swelling of the body?

Yes No

Doesn't know

Refused to answer

Id10254 - Did she have any lumps or sores in the mouth?

Yes No

Doesn't know

Refused to answer

Id10253 - Did she have lumps anywhere else on the body?

*Lumps can be in the neck, armpit, groin or other areas of the body.*

Yes No

Doesn't know

Refused to answer

Id10255 - Did she have any lumps on the neck?

Yes No

Doesn't know

Refused to answer

Id10256 - Did she have any lumps on the armpit?

Yes No

Doesn't know

Refused to answer

Id10257 - Did she have any lumps on the groin?

Yes No

Doesn't know

Refused to answer

Id10258 - Was she in any way paralysed?

Yes No

Doesn't know

Refused to answer

Id10259 - Did she have paralysis of only one side of the body?

Yes No

Doesn't know

Refused to answer

Id10260 - Did she have paralysis of both legs?

Yes No

Doesn't know

Refused to answer

Id10261 - Was there difficulty or pain in swallowing?

Yes No

Doesn't know

Refused to answer

Id10262_units - For how long did she have difficulty or pain in swallowing?

*If the respondent is unable to answer, prompt: Did the difficulty or pain in swallowing last for less than 1 week interviewer to enter 6 days, or for at least 1 week interviewer to enter 8 days?*

Days Week Months

Doesn't know

Refused to answer

Id10262_a - Enter how long before death she had difficulty or pain in swallowing in days:

*Enter 0-30 days. Less than 1 day or 24 hours = 0 days; 1 week = 7 days.*

Id10262_b - Enter how long before death she had difficulty or pain in swallowing in months:

*Enter 1-60 months.*

Id10262_c - Did swallowing become impossible?

Yes No

Doesn't know

Refused to answer

Id10265 - Did she have yellow discoloration of the eyes?

Yes No

Doesn't know

Refused to answer

Id10266_units - For how long did she have the yellow discoloration?

*If the respondent is unable to answer, prompt: Did the yellow discoloration last for less than 3 weeks interviewer to enter20 days, or for at least 3 weeks interviewer to enter 22 days? Enter 1 unit only: 0-30 days or 1-60 months. Less than 1day or 24 hours = 0 days; 1 week = 7 days.*

Days Week Months

Doesn't know

Refused to answer

Id10266_a - Enter how long she had the yellow discoloration in days:

*Enter 0-30 days. Less than 1 day or 24 hours = 0 days; 1 week = 7 days.*

Id10266_b - Enter how long she had the yellow discoloration in months:

*Enter 1-60 months.*

Id10267 - Did her hair change in colour to a reddish or yellowish colour?

Yes No

Doesn't know

Refused to answer

Id10268 - Did she look pale or have pale palms, eyes or nail beds?

*Long term deficiency of the blood results in a pale, whitish appearance of the lips, tongue, and eye sac. Sometimes it is referred to as thinning or lack of blood, or pallor.*

Yes No

Doesn't know

Refused to answer

Id10485 - Did she suffer from extreme fatigue (always tired)?

*Probe whether the deceased felt so tired that she found it hard to get out the bed and do the routine things like taking a shower or changing clothes*

Yes No

Doesn't know

Refused to answer

Id10486 - Did she experience a new loss, change or decreased sense of smell or taste?

Yes No

Doesn't know

Refused to answer

# Signs and symptoms associated with pregnancy and women

Id10294 - Did she have any lumps and/or ulcers (wound) in the breast?

Yes No

Doesn't know

Refused to answer

Id10296 - Did she ever have a period or menstruate?

*If the answer is "NO", please ensure that there was no chance the deceased had recently been pregnant.*

Yes No

Doesn't know

Refused to answer

Id10299_a - Did she make use of contraceptive?

Yes No

Doesn't know

Refused to answer

Id10299 - Did her menstrual period stop naturally because of menopause?

Yes No

Doesn't know

Refused to answer

Id10300 - Did she have vaginal bleeding after cessation of menstruation?

Yes No

Doesn't know

Refused to answer

Id10301 - Was there excessive vaginal bleeding in the week prior to death?

*Important is the excessive quantity of blood*

Yes No

Doesn't know

Refused to answer

Id10302 - At the time of death was her period overdue?

Yes No

Doesn't know

Refused to answer

Id10303 - For how many weeks had her period been overdue?

*If the respondent is unable to answer, prompt: Was the period overdue for less than 4 weeks interviewer to enter 3 weeks or for at least 4 weeks interviewer to enter 5 weeks? Less than 1 week=0. 7 days=1 week. Enter "99" for "don't know. “Enter "88" for "refuse."*

Id10305 - Was she pregnant and not yet in labour at the time of death?

*A "Yes" response to this question means a foetus or baby remained in the mother's body after she died. If she was already in labour or actively aborting - please answer "NO" to Id10305.*

Yes No

Doesn't know

Refused to answer

Id10312 - Did she die during labour or delivery?

*A "Yes" response to this question excludes women dying during abortion or miscarriage.*

Yes No

Doesn't know

Refused to answer

Id10313 - Did she die after delivering a baby?

*Note that a maternal death is relevant up to 1 year after delivering a baby.*

Yes No

Doesn't know

Refused to answer

Id10314 - Did she die within 24 hours after delivery?

*-*

Yes No

Doesn't know

Refused to answer

Id10306 - Did she die within 6 weeks after delivery?

*-*

Yes No

Doesn't know

Refused to answer

Id10334 - Did she have a pregnancy that ended in an abortion or miscarriage within 6 weeks before her death?

*-*

Yes No

Doesn't know

Refused to answer

Id10333 - Did she attempt to terminate the pregnancy?

Yes No

Doesn't know

Refused to answer

Id10308 - Did she die less than 1 year after delivery, abortion or miscarriage?

Yes No

Doesn't know

Refused to answer

Id10310 - Please confirm that in the 12 months prior to her death, the woman was not pregnant, she did not have a delivery, and she also did not have an abortion or miscarriage.

*Note: If this was a maternal death, please go back to indicate the correct circumstances.*

OK

Id10304 - Did she have a sharp abdominal (tummy) pain in the first 3 months of pregnancy?

Yes No

Doesn't know

Refused to answer

Id10304_a - Did she faint when she had the sharp abdominal (tummy) pain?

Yes No

Doesn't know

Refused to answer

# Questions about possible maternal deaths

Id10309 - For how many months was she pregnant?

*If the respondent is unable to answer, prompt: Was she pregnant for less than 6 months interviewer to enter 5 months or for more than 6 months interviewer to enter 7 months? For don't know, enter "99." For refused, enter "88."*

Id10317 - How many babies was she pregnant with?

Singleton Twins

Triplets or more Don't know

Refused to answer

Id10321_a - Did she suffer from high blood pressure before pregnancy?

Yes No

Doesn't know

Refused to answer

Id10321 - During pregnancy, did she suffer from high blood pressure?

Yes No

Doesn't know

Refused to answer

Id10322_a - Did she have foul smelling vaginal discharge during pregnancy?

Yes No

Doesn't know

Refused to answer

Id10325 - Did bleeding occur while she was pregnant?

Yes No

Doesn't know

Refused to answer

Id10327 - Was there vaginal bleeding during the last 3 months of pregnancy but before labour started?

*The last 3 months of pregnancy refers to the 7th-9th months of a full term pregnancy.*

Yes No

Doesn't know

Refused to answer

Id10323 - Did she suffer from convulsions during the last 3 months of pregnancy and/or after delivery?

Yes No

Doesn't know

Refused to answer

Id10324 - Did she have blurred vision during the last 3 months of pregnancy and/or after delivery?

*The last 3 months of pregnancy refers to the 7th-9th months of a full term pregnancy.*

Yes No

Doesn't know

Refused to answer

Id10328 - Did she have excessive bleeding during labour or delivery?

*Here the excessive quantity of blood DURING birth is what we ask for*

Yes No

Doesn't know

Refused to answer

Id10329_a - Did she have excessive bleeding after delivery?

*Here the excessive quantity of blood AFTER birth is what we ask for*

Yes No

Doesn't know

Refused to answer

Id10329_b - Did she have excessive bleeding during or after abortion or miscarriage?

*Here the excessive quantity of blood AFTER abortion or miscarriage is what we ask for*

Yes No

Doesn't know

Refused to answer

Id10322_b - Did she have foul smelling vaginal discharge after delivery/abortion?

Yes No

Doesn't know

Refused to answer

Id10331 - Did she deliver or try to deliver an abnormally positioned baby?

*Enquire the respondent about his/hers understanding of what is an abnormally positioned baby; if unclear or wrong, explain that it refers to baby’s whose first body part exiting the vagina is not the head.*

Yes No

Doesn't know

Refused to answer

Id10332 - For how many hours was she in labour?

*If the respondent is unable to answer, prompt: Was she in labour for less than 24 hours interviewer to enter 23 hours, or for more than 24 hours interviewer to enter 25 hours. Less than 60 minutes = 0 hours. 1 day=24 hours. Enter "99"for "don't know." Enter "88" for "refuse."*

# How did the mother deliver her baby?

Id10342 - Was the delivery normal vaginal, without forceps or vacuum?

Yes No

Doesn't know

Refused to answer

Id10343 - Was the delivery vaginal, with forceps or vacuum?

Yes No

Doesn't know

Refused to answer

Id10344 - Was the delivery a Caesarean section?

Yes No

Doesn't know

Refused to answer

Id10330 - Was the placenta completely delivered?

Yes No

Doesn't know

Refused to answer

Id10337 - Where did she give birth?

Home (place of residence) Private hospital

Public hospital Maternity home

Traditional birth attendant Religious house

On route to hospital or facility Others please specify

Doesn't know

Refused to answer

Other Please Specify

Id10319 - How many births, including stillbirths, did she/the mother have before this pregnancy?

*For don't know, enter "99." For refused, enter "88."*

Id10320 - Had she had any previous Caesarean section?

Yes No

Doesn't know

Refused to answer

Id10340 - Did she have an operation to remove her uterus shortly before death?

*Question is relevant for cases of obstructed labour and ruptured uterus.*

Yes No

Doesn't know

Refused to answer

# Risk factors

Id10411 - Did she drink alcohol?

Yes No

Doesn't know

Refused to answer

Id10413 - Did she ever smoke tobacco?

*To clarify, the series inquires about tobacco consumption about any period during life i.e. not only the current status before death.*

Yes No

Doesn't know

Refused to answer

Id10413_a - For how long did she smoke tobacco?

*If deceased smoked for less than 1 month, enter 1 month as duration for the VA interview.*

Yes No

Doesn't know

Refused to answer

Id10413_d - How many months/years

Id10413_b - Did she ever smoke daily?

*The question intends to know if there was ever a period in the life of the deceased when she was smoking daily - even if it was not continuous or if the deceased was not smoking in the period leading to death.*

Yes No

Doesn't know

Refused to answer

d10414 - Did she ever chew and/or sniff tobacco?

*To clarify, the series inquires about tobacco consumption about any period during life i.e. not only the current status before death.*

Yes No

Doesn't know

Refused to answer

Id10414_a - For how long did she chew and/or sniff tobacco?

Yes No

Doesn't know

Refused to answer

Id10414_d - How many months/years

Id10414_b - Did she ever chew and/or sniff tobacco daily?

*The question intends to know if there was ever a period in the life of the deceased when she was chewing and/or sniffing tobacco daily - even if it was not continuous or if the deceased was not chewing and/or sniffing in the period leading to death.*

Yes No

Doesn't know

Refused to answer

# Health service utilisation

Id10417- Did she register for antenatal clinic?

Yes No

Doesn't know

Refused to answer

Id10417_a - Did she attend antenatal clinic?

Yes No

Doesn't know

Refused to answer

Id10417_b - Please specify the antenatal clinic/facility

Id10417_c - Why didn't she attend antenatal clinic ?

Id10418 - Did she receive any treatment for the illness that led to death?

Yes No

Doesn't know

Refused to answer

Id10419 - Did she receive oral rehydration salts?

Yes No

Doesn't know

Refused to answer

Id10420 - Did she receive (or need) intravenous fluids (drip) treatment?

Yes No

Doesn't know

Refused to answer

Id10421 - Did she receive or need a blood transfusion?

Yes No

Doesn't know

Refused to answer

Id10422 - Did she receive or need treatment/food through a tube passed through the nose?

Yes No

Doesn't know

Refused to answer

Id10423 - Did she receive or need injectable antibiotics?

*Injectable antibiotics exclude immunisations, vaccines and pain killers. Antibiotics are given against infection i.e., germs.*

Yes No

Doesn't know

Refused to answer

Id10424 - Did she receive or need antiretroviral therapy ART?

Yes No

Doesn't know

Refused to answer

Id10425 - Did she have or need an operation for the illness?

Yes No

Doesn't know

Refused to answer

Id10426 - Did she have the operation within 1 month before death?

Yes No

Doesn't know

Refused to answer

Id10435 - Did a health care worker tell you the cause of death?

Yes No

Doesn't know

Refused to answer

Id10436 - What did the health care worker say?

# Civil registration numbers - Civil registration: "This refers to the legal death

certificate obtained from the civil registration authorities show image of local death certificate if available."

Id10069_a - Do you have a Death Certificate from the Civil Registry?

Yes No

Id10070 - Death registration number/certificate

*Enter a "-" if this information is not available.*

Id10071_check - Is the date of registration available?

Yes No

Id10071 - Date of registration

Id10072 - Place of registration

*Enter a "-" if this information is not available.*

Id10073 - National identification number of deceased

*Record the National Identification Number. For newborns that have no ID number, use the mother's ID. If the mother's ID is not available, use the father's ID. If this information is unknown or not available, enter "-". Note whose ID was entered in the blank after the ID has been recorded.*

# Medical certificate of cause of death - Death certificate with cause of death: "This refers to the medical certificate of cause of death show image of local medical certificate of cause of death if available."

Id10462 - Was a medical certificate of cause of death issued?

*The following information serves only to complete cause of death information in some environments. In routine CRVS this information could be skipped in the interview, and information be collected from other sources, if available.*

Yes No

Doesn't know

Refused to answer

Id10463 - Can I see the medical certificate of cause of death?

*This section aims to collect information from the international standard medical certificate of cause of death. This level of detail may or may not be present in the death certificate issued to the family. Record "no" if medical information about the cause of death is not available. The medical certificate of cause of death is commonly obtained from a physician at a hospital and should be distinguished from the Death Certificate which is issued by the civil registration organisation.*

Yes No

Doesn't know

Refused to answer

Id10464 - Record the immediate cause of death from the certificate line 1a

*If this detail is not present, record "-" not available.*

Id10465 - Duration of the immediate cause of death Is:

*For all following lines, add duration, if stated. If this detail is not present, record "-" not available.*

Id10466 - Record the first antecedent cause of death from the certificate line 1b

*An antecedent cause is the one that caused the one on the line above, e.g. diabetes mellitus may be an antecedent cause to kidney disease. If this detail is not present, record "-" not available.*

Id10467 - Duration of the first antecedent cause of death Is:

*If this detail is not present, record "-" not available.*

Id10468 - Record the second antecedent cause of death from the certificate line 1c

*If this detail is not present, record "-" not available.*

Id10469 - Duration of second antecedent cause of death Is:

*If this detail is not present, record "-" not available.*

Id10470 - Record the third antecedent cause of death from the certificate line 1d

*If this detail is not present, record "-" not available.*

Id10471 - Duration of third antecedent cause of death Id:

*If this detail is not present, record "-" not available.*

Id10472 - Record the contributing causes of death from the certificate part 2

*If this detail is not present, record "-" not available.*

Id10473 - Duration of the contributing causes of death part2:

*If this detail is not present, record "-" not available.*

Id10481 Inform the respondent that the VA interview has come to an end. Thank the respondent for their time and answers, and ask if the respondent has any questions or comments to make. Use this section to record any additional details you and/or the respondent have about the interview.

OK
