# Supplementary material for: Adapting the 2022 WHO verbal autopsy tool for use in Lagos State, Nigeria: insights from the LVASA-SRS project
Source: BMC Res Notes. 2025 Dec 29;18:508. doi: 10.1186/s13104-025-07560-1 (PMC12751495; doi:10.1186/s13104-025-07560-1)
Supplement: Supplementary file 2 — Supplementary Material 2. [file 13104_2025_7560_MOESM2_ESM.docx]

**Additional file 2: Summary of skip logic and questionnaire modifications**

| **#** | **Item/Block (ID)** | **What was skipped/reordered/changed** | **Reason** | **Where it went / Current status** |
| --- | --- | --- | --- | --- |
| 1 | Id10019 Sex of deceased | Constrained to Female only (removed Male, Ambiguous/Intersex) | Maternal deaths/stillbirths scope of the LVASA project | Option set restricted; not moved |
| 2 | Age-group branching | Removed child/neonate/adult branching | Non-maternal items irrelevant | Removed |
| 3 | Interview language | Replaced “Language 2/3” with English, Yoruba, Pidgin | Most common localized language in Lagos | Localized choices; not moved |
| 4 | Consent/time stamps (Id10011, Id10481) | Start/end time fields omitted; Consent timestamp handled by the Kobo Toolbox in the backend, immediately interview starts | Streamlining; device metadata | Start/end time removed; consent retained |
| 5 | Id10051 demographic option | Removed “need to collect” toggle; | Should be simplified and ensure consistency | Toggle removed; subset kept inline |
| 6 | Id10059 marital status | Localized options; removed “Too young” | Maternal context, clarity | Options revised; same position |
| 7 | Id10058/Id10337 place categories | Localized facility list (Home, Private, Public, TBA, etc.) | Nigerian context; maternal relevance | Lists localized; same concept |
| 8 | Adult chronic diagnosis (COPD, dementia, depression) | Retained | Maternal diagnostic item still relevant | Retained |
| 9 | Id10130 dengue diagnosis | Removed | Low epidemiologic relevance | Removed |
| 10 | Id10141 stroke; Id10133 heart disease | Retained | Important maternal diagnosis | Retained in Medical history |
| 11 | Injuries section (Id10077–Id10077_b) | Retained ≤7-day; maternal block still administered | Maternal context needed even with injury | Retained; no truncation of maternal section |
| 12 | Dates (Id10012, Id10021, Id10023_a, Id10024, Id10071) | Reformatted to yyyy-mm-dd; added “Age in years” | Data quality/Kobo Toolbox compatibility | Reformatted; not moved |
| 13 | COVID exposure/testing (Id10482–Id10487) | Retained | Respiratory/maternal | Retained |
| 14 | Narrative (Id10476–Id10477) | Retained; keywords slightly tuned | Supports COD algorithm | Retained |
| 15 | Duration units (multiple IDs) | Added “Weeks” to several duration items; Id10216 units broadened | Usability/recall accuracy | Reframed within same items |
| 16 | Id10199 abdominal pain location | Added “Mid abdomen” | Better layman description | Expanded options; same item |
| 17 | Id10235 rash location wording | Clarified body regions | Layman understanding | Wording updated; same item |
| 18 | Maternal module order | Maternal history moved earlier in symptom flow | Prioritise maternal timelines; reduce fatigue | Reordered earlier; content retained |
| 19 | Menopause/PMB (Postmenopausal bleeding); (Id10299–Id10301) | Routing simplified; items retained | Distinguished PMB vs pregnancy bleeding | Retained; simpler routing |
| 20 | Id10310 maternal confirmation | Replaced complexity with single “OK/Confirm” | Field simplicity | Single confirmation |
| 21 | Maternal risk features block | Retained and tightly grouped | Core maternal COD indicators | Retained; regrouped |
| 22 | Education/literacy (Id10063–Id10064) | Retained without changes | Equity/socio-demographic needs | Retained |
| 23 | Occupation free text (Id10066) | Removed | Streamlined; economic status sufficient | Removed |
| 24 | Tobacco chew/sniff (Id10414 series) | Removed | Low yield for maternal COD; help shorten interview | Removed |
| 25 | Smoking duration/intensity (Id10413_a/_d/_b) | Follow-ups removed; basic kept | Reduce time; limited impact | Kept Yes/No; removed follow-ups |
| 26 | Health service use (Id10418–Id10426) | Retained core items | Maternal care pathway relevance | Retained |
| 27 | CRVS & MCCD (Id10069_a–Id10073; Id10462–Id10473) | Retained with ability to skip if unavailable | Programmatic linkage | Retained (conditional on availability) |
